# Environmental dependency of amphibian–ranavirus genotypic interactions: evolutionary perspectives on infectious diseases

**DOI:** 10.1111/eva.12169

**Published:** 2014-06-16

**Authors:** Pierre Echaubard, Joel Leduc, Bruce Pauli, V Gregory Chinchar, Jacques Robert, David Lesbarrères

**Affiliations:** 1 Department of Biology, Genetics and Ecology of Amphibians Research Group (GEARG), Laurentian UniversitySudbury, ON, Canada; 2 Science and Technology Branch, National Wildlife Research Centre, Environment Canada, Carleton UniversityOttawa, ON, Canada; 3 Department of Microbiology, University of Mississippi Medical CenterJackson, MS, USA; 4 Department of Microbiology and Immunology, University of Rochester Medical CenterRochester, NY, USA

**Keywords:** genotype–genotype–environment interactions, host–pathogen interactions, *Lithobates pipiens*, *Lithobates sylvaticus*, ranavirus.

## Abstract

The context-dependent investigations of host–pathogen genotypic interactions, where environmental factors are explicitly incorporated, allow the assessment of both coevolutionary history and contemporary ecological influences. Such a functional explanatory framework is particularly valuable for describing mortality trends and identifying drivers of disease risk more accurately. Using two common North American frog species (*Lithobates pipiens* and *Lithobates sylvaticus*) and three strains of frog virus 3 (FV3) at different temperatures, we conducted a laboratory experiment to investigate the influence of host species/genotype, ranavirus strains, temperature, and their interactions, in determining mortality and infection patterns. Our results revealed variability in host susceptibility and strain infectivity along with significant host–strain interactions, indicating that the outcome of an infection is dependent on the specific combination of host and virus genotypes. Moreover, we observed a strong influence of temperature on infection and mortality probabilities, revealing the potential for genotype–genotype–environment interactions to be responsible for unexpected mortality in this system. Our study thus suggests that amphibian hosts and ranavirus strains genetic characteristics should be considered in order to understand infection outcomes and that the investigation of coevolutionary mechanisms within a context-dependent framework provides a tool for the comprehensive understanding of disease dynamics.

## Introduction

Despite a century of relatively efficient control and prevention, the last three decades have witnessed the increasing emergence and re-emergence of infectious diseases (Cohen 2000). Emerging infectious diseases (EIDs) are caused by pathogens that have recently increased in incidence, expanded their host or geographic range, have been newly discovered, or are caused by a newly evolved agent (Daszak et al. 2000). The emergence of viruses, such as Nipah, Hendra, SARS, and avian influenza A to name a few, has increased scientific and public awareness regarding the threats of infectious diseases and their possible detrimental consequences for biodiversity, ecosystem functioning, and public health (Daszak et al. 2000; Binder et al. 1999; Morens et al. 2004). Historically, human EIDs have received more attention than their wildlife counterparts. However, the increasing occurrence of pathogens crossing the barriers between their natural reservoirs to human populations (zoonosis), has boosted research on wildlife diseases (Antia et al. 2003). In addition, emerging pathogens can have a significant influence on wildlife population dynamics (Keesing et al. 2006; Pedersen and Fenton 2007) by altering community functioning (Hudson and Greenman 1998). Diseases also contribute to species extinctions (MacPhee and Greenwood 2013) especially in cases where infections occur synergistically with habitat destruction and reduce populations below a critical size threshold for survival (De Castro and Bolker 2005).

Despite the critical importance of understanding patterns of disease emergence, spread and severity, our ability to do so is often limited by the complexity of the interactions among various factors (Plowright et al. 2008). For example, ranaviruses, known to infect at least 72 amphibian species in 14 families (Miller et al. 2011) have caused amphibian die-offs on five continents (Gray et al. 2009). Yet, few common features have been found to predict the extent of morbidity or mortality (Lesbarrères et al. 2012). Elucidating the mechanisms of ranavirus induced mortality in wild amphibian populations is difficult because amphibian susceptibility and ranavirus infectivity may vary depending on host–virus combinations. For instance, susceptibility to ranaviruses differs among populations within species and across phylogenetic lineages (Hoverman et al. 2010; Schock et al. 2008). In African clawed frogs (*Xenopus laevis*), the amount of genetic variability in MHC genes appears to be correlated with ranavirus susceptibility (Gantress et al. 2003). From the pathogen perspective, induced damage (sensu Casadevall and Pirofski 1999) may differ with the species, strain or isolate of ranavirus involved in the infection (Echaubard 2012). For instance, at least 30 isolates of *Ranavirus* have been mapped phylogenetically, with over half infecting amphibians (Hyatt et al. 2000; Wang et al. 2003), and genetically unique isolates may represent novel ranavirus types that have enhanced virulence (Ridenhour and Storfer 2008).

The large amount of variation in susceptibility among amphibian hosts and in pathogenicity among ranavirus strains suggests that host and virus may coevolve in response to each other, leading to local coadaptations through genotype × genotype interactions (Thompson 2005). In a system where environmental influences are absent, the outcome of infection in a given host–parasite interaction would be determined by the host and parasite genotypes (see Lambrechts et al. 2006 for a discussion). Practically, this means that no parasite is best at infecting all hosts, and no host is best at resisting all parasites, so that the result of an infection depends on the specific combination of the two protagonists. Investigation of genotype × genotype interactions in the amphibian-ranavirus system and the identification of coevolutionary trends seem particularly appropriate and promising as it may reveal subtle mechanisms responsible for unexplained variations in disease outbreaks and severity (Miller et al. 2011).

In addition, environmental change may further modulate patterns of host susceptibility and/or pathogen virulence, revealing the context-dependent nature of disease dynamics (Echaubard et al. 2010; Daskin and Alford 2012). For instance, environmental attributes related to the geographic locations of populations may be a factor influencing amphibian hosts susceptibility to ranavirus. Watersheds located at higher elevations have been associated with increased probability of ranavirus infection and epizootics in some North American amphibian populations (Gahl and Calhoun 2008). Environmental conditions such as ambient temperature or pollution have also been associated with increased prevalence of ranavirus infection and disease (Balseiro et al. 2010; Kerby et al. 2011). The influence of temperature on host and parasite has been increasingly documented as a critical environmental feature modulating host–pathogen interactions outcomes (Wolinska and King 2009; Vale and Little 2009). For instance, while increasing temperature may help hosts to resist and eliminate pathogens through enhancement of immunity, elevated temperatures may also promote the replication of pathogens such as viruses (Thomas and Blanford 2003; Rojas et al. 2005). Additionally, prolonged exposure to supra-optimal temperatures may be detrimental to host survival. Intense fever, for example, has been shown to be harmful to hosts when occurring for long period of time (Kluger et al. 1998). The relationship between temperature and host survival with regard to pathogen infection is therefore complex.

The investigation of genotype × genotype interactions in a context-dependent framework, where environmental factors (such as temperature) are explicitly incorporated, provides an ecological context to evolutionary determinants and will improve our understanding of epidemiological issues in wildlife. Despite its clear conceptual relevance, only few studies have investigated the three-way interaction between host genotype, pathogen genotype, and temperature in the context of infectious diseases (Tétard-Jones et al. 2007). In this study, we experimentally tested for genotype × genotype × temperature interactions by designing a fully factorial laboratory experiment using two common North American frog species and three ranavirus (FV3) strains in two temperature settings. We addressed the three following questions: (i) is the outcome of the interaction specific to a host–strain combination? (ii) does temperature influence pathogen infectivity and/or host mortality? (iii) does temperature modulate host–strain genotypic interactions resulting in genotype × genotype × temperature interactions?

## Material and methods

### Experimental organisms

The genus *Ranavirus* (family *Iridoviridae*) is composed of six viral species recognized by the ICTV (Jancovich et al. 2012) all of them infecting poikilotherm vertebrates including fish, reptiles, and amphibians (Chinchar et al. 2009). Most of what is known about ranavirus replication is based on studies of frog virus 3 (FV3) the type species of the genus *Ranavirus*. Amphibians are most vulnerable to FV3 infection during the larval or early metamorphic stages of development, and mortality of infected animals usually occurs during these developmental stages while adults are relatively resistant owing to more competent immune function (Robert et al. 2005). Effects of ranavirus infection on larvae can sometimes be seen externally as skin ulcerations or systemic hemorrhaging. When no external signs of infection are visible, more subtle symptoms, such as erratic swimming, lethargy or lack of equilibrium, may indicate morbidity due to infection in larvae (Gray et al. 2009). Three different FV3-like viruses were chosen for this study: (i) the original FV3 isolate (wt-FV3) that was first isolated in 1965 (Granoff et al. 1965) is known to infect both *Lithobates pipiens* and *L. sylvaticus* (Duffus et al. 2008). (ii) a more recent variant (azacR) that is defective in its ability to methylate the viral genome and may trigger a TLR-9 response, might be the least virulent, and (iii) an FV3-like isolate designated Spotted salamander Maine Virus (SsMeV, US National Wildlife Health Center #15919-03 + 08) which was isolated during an outbreak involving frogs and salamanders from Connor Township, Aroostook County, Maine in July 1998. Nucleotide sequence analysis of the major capside protein gene (MCP) suggests that SsMeV is an FV3-like virus and recent 454 GS-FLX sequencing suggests that SsMeV is 98.79% identical to FV3 at the nucleotide level but differ in several Open Reading Frame (ORF) coding regions possibly involved in infectivity (Morrison et al. 2014). The choice of SsMev was also supported by a recent study showing that Ranaviruses are prone to jump from species to species (Bayley et al. 2013). The rationale for choosing azacR, wt-FV3, and SsMeV was thus to have isolates with variable presumed infectivity (a situation likely to occur in nature) and consequently to increase the likelihood of observing variation in infection outcomes and to reveal genotypic interactions. All FV3 isolates were propagated on fathead minnow cells at the University of Mississipi Medical Center (Jackson, MS, USA).

The Northern leopard frog (*L. pipiens*) and the wood frog (*L. sylvaticus*) are two common North American frog species known to be highly susceptible to ranavirus. Mass die-offs and local population declines associated with ranaviral disease have been documented in both species (Greer et al. 2005). While using different habitats during the summer months (i.e., grasslands for the Northern leopard frog, woodlands for the wood frog), both species can be found spawning in the same wetlands during the spring months, suggesting the possibility for horizontal transmission of ranavirus to occur, in turn underlying the relevance of using these two species as hosts in our study. In July 2010, we received tadpoles, approximately Gosner stage 25 (Gosner 1960), of *L. pipiens* and *L. sylvaticus* from the Environment Canada Atlantic Laboratory for Environmental Testing in Moncton, NB., Canada (courtesy of Paula Jackman). Tadpoles were kept separate by species and from the initial egg masses they hatched from. *L. pipiens* tadpoles originated from a single egg mass collected in eastern Ontario while *L. sylvaticus* tadpoles originated from two distinct egg masses collected in New Brunswick. Egg masses were collected immediately after oviposition hence reducing environmental influence on embryos development.

### Experimental design and procedures

To investigate variability in the interactions between *Lithobates sp*. hosts and ranavirus strains, we designed a full factorial experiment in which tadpoles from each of three egg masses were separately exposed to all three ranaviruses and a control (no infection) resulting in 6 and 12 possible host species and host genotype-ranavirus strains combinations respectively, each replicated three times. Every treatment consisted of 12 Gosner stage 25 (Gosner 1960) *L. pipiens* or *L. sylvaticus* tadpoles. In addition, a temperature treatment (14°C and 22°C; Thermo Incubator Model 3740) representing a relevant range for species in similar latitudes (Laugen et al. 2003) was used to investigate the potential influence of temperature variation. A total of 420 tadpoles (140 of each genotype) were used in a nested design (host genotypes nested in host species) allowing us to discriminate the effect of host species versus host genotypes in driving infection outcomes along a gradient of genetic distances (phylogenetic versus genotypic).

In all treatments, tadpoles were placed for 12 h in 125 mL plastic vials (each replicate in a separate vial) containing either 50 mL of clean dechlorinated water (control) or 50 mL of water containing 10 000 PFU/mL of either wt-FV3, azacR, or SsMeV (Gantress et al. 2003). Tadpoles were then moved together with the contaminated water into 2 L plastic containers containing 1 L of dechlorinated water (preaged for 3 days) for the remainder of the experiment. Starting at week 3, 100% of the water in each tank was replaced weekly with clean dechlorinated aged (24 h) water. As a result, exposed tadpoles were held in virus-containing water for 3 weeks, a period long enough for tadpoles to be in close proximity with residual infection (Echaubard et al. 2010).

The host density (number of tadpoles per volume of water) was adjusted to three tadpoles per 250 mL of water to minimize effect of density on tadpole development (Echaubard et al. 2010). Volume of water was adjusted after each death to keep density similar from the beginning to the end of the experiment. Tadpoles were fed on a weekly basis with standard tadpole food (Carolina Biological Supply Company, Burlington, NC) at 30 mg/tadpole for week 1, 60 mg/tadpole for week 2, and 120 mg/tadpole for week 3 until the end of the experiment. Food was administered to each tank after each weekly water change. Tanks were inspected on a daily basis for mortality. Dead tadpoles were removed using disposable plastic pipettes to prevent any scavenging by survivors. Dead individuals were kept in separate plastic vials filled with ethanol and stored at −25°C for subsequent analyses. The experiment terminated when all individuals died or reached metamorphosis. Metamorphs were killed using MS-222 following the procedures described in protocol #2010-04-02 approved by the Laurentian University Animal Care Committee.

To monitor for ranavirus infection, livers were removed from all animals (including euthanized metamorphs), crushed in a 1.5 mL Eppendorf tube for DNA extraction (QIAmp DNeasy Kit; Qiagen, Mississauga, Canada). After extraction, a double-blind PCR was performed using primers and conditions known to successfully amplify a portion of the FV3 major capsid protein gene: MCP-ranavirus-F (5′-GACTTGGCCACTTATGAC-3′) and MCP-ranavirus-R (5′- GTCTCTGGAGAAGAAGAA; Mao et al. 1997). Only individuals showing two positive amplifications were considered infected.

### Statistical analyses

We investigated the effects of four independent factors (host species, host genotype, ranavirus strain, and temperature), and their interactions, on mortality and infection patterns. Interactions of interest were between ranavirus strain and host species/genotype, temperature and host *or* ranavirus strain and between temperature, host and ranavirus strain. All the analyses shared a common design where temperature, species and strain factors were fixed and host genotypes and treatment replicates were included as random factors. Host genotypes were nested into host species to avoid pseudo-replication and to discriminate species and host genotype effects. Tank was also used as a block factor.

Host mortality trends were analyzed using the Cox proportional-hazards regression model with coefficients estimated using maximum likelihood. The significance of the predictors was assessed using the Wald statistic, the complement (1-KM) of the Kaplan–Meier (KM) was used to estimate mortality probabilities. Individuals surviving to the end of the experiment were censored to account for a lack of information about their true time to death. Censoring is a standard technique that down-weights the influence of these individuals in the survival analysis (Leung et al. 1997).

Infection patterns were analyzed using a logistic regression model with binomial error distribution and logit link function. To select the most appropriate explanatory model for the logistic regression, a preliminary analysis of deviance was performed during which predictors and their interactions where added sequentially until residual deviance was minimized. The estimates of the coefficients and the intercepts of the selected logistic regression model were calculated via maximum-likelihood estimation and significant effects were assessed using *z*-scores.

All statistical tests were performed using ‘r’ version 3.0.1 (The R Core Team 2013); packages ‘Survival’ and ‘KMsurv’ were used for survival analysis.

## Results

### Infection

**Figure 1:**
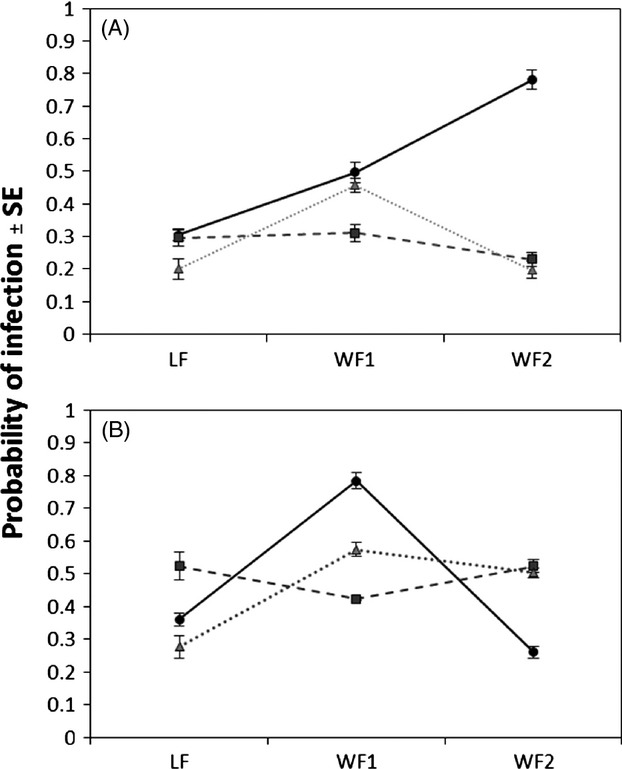
Interaction plot showing average probability of infection (±SE) for all nine combinations of the three host genotypes and three ranavirus strains (azacR = solid line and circles; SsMeV = dotted line and triangles; wt-FV3 = dashed line and squares; *n* = 36 ± 3.09 on average per combination), at 22°C (A) and 14°C (B).

PCR monitoring indicated that none of the control individuals were infected while the probability of infection in exposed individuals ranged between 0.23 and 0.78 (Fig. 1). A preliminary analysis of deviance was performed to select the model that best fit infection data and which retained all variables (temperature, genotypes nested within species, species, strains) and interactions (Residual deviance = −9.77e-15, AIC = 92.084).

In the selected model, temperature had a significant influence on the odds of infection. The probability of infection increased at low temperature (14°C). The average probability of infection was 0.47 at 14°C vs 0.36 at 22°C (df = 1, *Z* = −2.821, *P* = 0.004; Fig. 1). There were also significant effects related to the species (df = 1, *Z* = 2.790, *P* = 0.005) or the genotypes within species (df = 3, *Z* = −2.642, *P* = 0.008) on infection probability. On average, Northern leopard frogs (NLF) had a lower probability of infection (0.32) compared to the two wood frog (WF) genotypes (0.5 and 0.41 for WF1 and WF2, respectively; Fig. 1). While no significant differences among virus strains (df = 2, *Z* = −1.296, *P* = 0.195) was observed, individuals exposed to azacR tended to have the highest probability of infection (0.5), followed by wt-FV3 (0.38; df = 1, *Z* = −1.590, *P* = 0.112) and SsMeV (0.37; df = 1, *Z* = −1.857, *P* = 0.063; Fig. 1).

The main effects of temperature, strain, and species were strongly influenced by each other with significant statistical interactions among them. There was a significant temperature × species interaction (df = 3, *Z* = −2.3, *P* = 0.021) primarily driven by the higher probability for WF to be infected, especially at 22°C (differences NLF vs WF at 22°C: df = 1, *Z* = −3.465, *P* < 0.001; differences NLF vs WF at 14°C: df = 1, *Z* = −1.844, *P* = 0.065; Fig. 1). We also observed a significant temperature × strain interaction (df = 4, *Z* = 1.98, *P* = 0.048) with a higher probability for azacR to infect amphibian hosts at 22°C as compared to other strains (azacR vs wt-FV3: df = 1, *Z* = −2.211, *P* = 0.027; azacR vs SsMeV: df = 1, *Z* = −2.208, *P* = 0.027). Such a difference was no longer observed at 14°C (azacR vs wt-FV3: df = 1, *Z* = 0.498, *P* = 0.618; azacR vs SsMeV: df = 1, *Z* = −0.285, *P* = 0.775; Fig. 1). The probability of infection by a given strain was also dependent on the species (strain × species interaction; df = 4, *Z* = −2.534, *P* = 0.011). On average, NLF individuals had a lower chance to be infected by azacR as compared to WF (0.33 vs 0.58 for NLF and WF, respectively). Among WF genotypes, WF1 showed a higher infection probability than WF2 (0.64 vs 0.52 for WF1 and WF2, respectively). The extent of the differences among host genotypes was also influenced by temperature, resulting in significant temperature × host genotype × strain interactions (df = 17, *Z* = −2.672, *P* = 0.007) and temperature × host species × strain interaction (df = 11, *Z* = 1.967, *P* = 0.049). In particular, overall probability of infection by SsMeV increased from 0.29 at 22°C to 0.45 at 14°C, but the extent of this increase was contingent on the host genotype, as the probability of infection went from 0.2 to 0.27 in NLF and from 0.2 to 0.50 in WF2 at 22°C and 14°C, respectively. Similarly, the probability of infection by wt-FV3 went from 0.3 at 22°C to 0.52 at 14°C in NLF and from 0.23 at 22°C to 0.52 at 14°C in WF2 while in WF1 the extent of the increase was smaller (from 0.31 to 0.42 at 22°C and 14°C, respectively; Fig. 1).

### Mortality

The overall probability of death was higher in infected tadpoles than in controls (59% vs 38%; df = 1, W = 15.76, *P* < 0.001), and varied significantly depending on the temperature (df = 1, W = 12.28, *P* < 0.001). At 14°C, the probability of death of infected tadpoles was on average of 0.67 (0.58–0.77), whereas at 22°C the probability of death dropped to 0.51 (0.38–0.61) among the different host species, host genotypes, and ranavirus strain combinations. Control individuals had a lower probability of death than infected individuals both at 14°C (0.54 vs 0.71; df = 1, W = 22.08, *P* < 0.001) and 22°C (0.37 vs 0.56; df = 1, W = 22.45, *P* < 0.001).

The significant effects of host species and ranavirus strains showed that Northern leopard frogs (NLF) and wood frogs (WF) differed in overall susceptibility to ranavirus, while ranavirus strains differed in their overall virulence. The lowest probability of death for infected animals was observed for NLF (0.57) while WF individuals died with a probability of 0.62 (df = 1, W = 21.88, *P* < 0.001) averaged across the different ranavirus strains. Control individuals had a lower probability of death than infected individuals in both NLF (0.41 vs 0.55; df = 1, W = 7.57, *P* = 0.05) and WF (0.5 vs 0.66; df = 1, W = 61.48, *P* < 0.001). We also observed a significant effect of host genotypes since both WF clutches were characterized by slightly different average probability of death with 0.58 and 0.64 for WF1 and WF2, respectively (df = 2, W = 21.66, *P* < 0.001). With regard to the effect of ranavirus strain, wt-FV3 was associated with the highest mortality probability (0.62) followed by azacR and SsMeV (0.58 and 0.57 respectively, df = 2, W = 7.86, *P* = 0.019).

**Figure 2:**
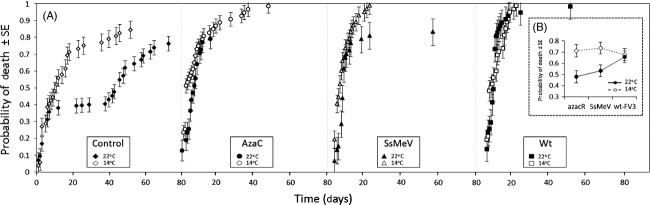
(A) Probability of death over time for azacR, SsMeV, wt-FV3 and control treatments at 14°C and 22°C illustrating strain × temperature interactions (*n* = 49 ± 3.72 on average per strain-temperature combination). (B) Interaction plot showing average probability of death (±SE) per strain-temperature combination. The complement of the Kaplan–Meier estimate was used to estimate the probability of death.

We also observed a significant strain × temperature interaction (df = 4, W = 22.89, *P* < 0.001; Fig. 2). At 14°C, tadpoles exposed to azacR, SsMeV, or wt-FV3 had a similar probability of death (df = 2, W = 2.17, *P* = 0.229) while at 22°C, tadpoles exposed to wt-FV3 had a higher probability of death than tadpoles exposed to SsMeV and tadpoles exposed to azacR (wt-FV3 vs SsMeV, df = 1, W = 45.04, *P* < 0.001; wt-FV3 vs azacR, df = 1, W = 43.97, *P* < 0.001; Fig. 2).

**Figure 3:**
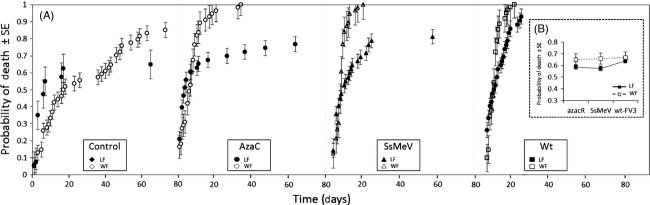
(A) Probability of death over time for leopard frog (LF) and wood frog (WF) tadpoles exposed to azaC, SsMeV and wt-FV3 illustrating strain × species interactions (*n* = 53 ± 7.01 on average per strain-species combination). (B) Interaction plot showing average probability of death (±SE) per strain-temperature combination. The complement of the Kaplan–Meier estimate was used to calculate the probability of death.

A significant interaction between ranavirus strain and host species for probability of death was also observed (df = 5, W = 29.76, *P* < 0.001; Fig. 3), with host species showing different patterns of mortality depending on which strain they have been exposed to. In particular, NLF tadpoles exposed to wt-FV3 displayed the highest probability of death as compared to the other strains (0.62 vs 0.56 for both azacR and SsMeV; df = 2, W = 7.96, *P* = 0.013). In WF, no differences among strains was observed (0.57 vs 0.61 and 0.60 for wt-FV3 and azacR, respectively; df = 2, W = 1.99, *P* = 0.247; Fig. 3).

**Figure 4:**
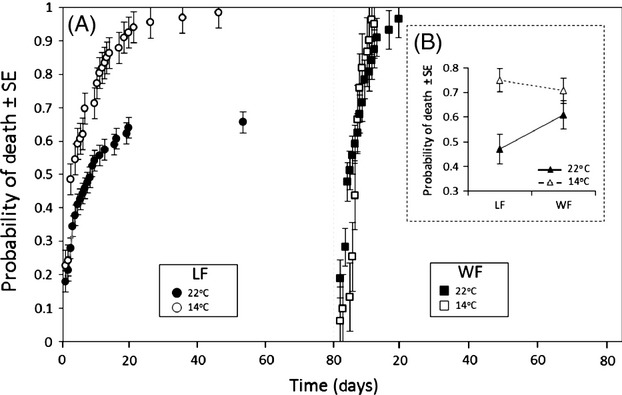
(A) Probability of death over time for northern leopard frog (LF) and wood frog (WF) individuals at 14°C and 22°C illustrating species × temperature interactions (*n* = 96 ± 4.73 on average per species-temperature combination). (B) Interaction plot showing average probability of death (±SE) per species-temperature combination. The complement of the Kaplan–Meier estimate was used to calculate the probability of death.

We also observed a significant host species × temperature interaction (df = 3, W = 39.84, *P* < 0.001; Fig. 4). At 22°C, while no difference in mortality was observed between WF hosts (WF1 vs WF2, df = 1, W = 0.5, *P* = 0.471), significantly fewer NLF tadpoles died (0.45) than WF tadpoles (0.54; df = 1, W = 23.76, *P* < 0.001; Fig. 4). At 14°C, however, no difference in mortality was observed among the two host species (df = 2, W = 2.77, *P* = 0.243).

Finally, we noted a significant strain × species × temperature interaction for probability of death (df = 11, W = 2.39, *P* = 0.034). For NLF, probability of death increased significantly from 0.45 at 22°C to 0.69 at 14°C, but the extent of this increase was contingent of the strain responsible for their infection. NLF probability of death went from 0.38 at 22°C to 0.74 at 14°C with azacR and from 0.36 at 22°C to 0.72 at 14°C with SsMeV while this increase was much smaller with wt-FV3 (from 0.6 at 22°C to 0.64 at 14°C). For WF, the probability of death increased at 14°C but the pattern of change was similar for all strains.

## Discussion

### Genotypic interactions between hosts and strains

Overall, our study supports species-dependent susceptibility to ranavirus in amphibians (Schock et al. 2008; Hoverman et al. 2010) and suggests that different ranavirus strains present different virulence. More importantly, our study also demonstrates that the impact of ranavirus is not only related to ranavirus strains and host species but is dependent on which genotypes interact with each other. These results highlight the importance of genotype × genotype interactions in host–pathogen systems and further underline the need for an eco-evolutionary framework to be used in infectious disease research.

The strains used in our experiment are closely related FV3 viruses (Essani et al. 1987; Morrison et al. 2014). Despite genomic similarity, we observed significant differences in the mortality that these different strains induced in their hosts, suggesting significant functional differences among them. Overall, wt-FV3 was associated with the highest probability of death, followed by SsMeV and azacR. However, azacR tended to be associated with the highest probability of infection, especially in warm conditions despite its low probability of induced mortality suggesting that higher infection rates are needed for azacR to induce the same mortality rates as wt-FV3. Because azacR cannot methylate its genomic DNA, it is thought to trigger a more vigorous pro-inflammatory response than parental wt-FV3 and hence show lower morbidity and mortality. In contrast, SsMeV showed a relative moderate virulence when mortality and infection probabilities were considered together. Recent sequence analysis revealed that SsMeV, although displaying an overall high level of identity to FV3, is the most divergent among the three strains used in this study with multiple amino acid deletions in putative functional genes involved with virulence (Morrison et al. 2014). Together, our results thus suggest that closely related ranavirus strains are able to induce variable degrees of mortality and infection in their amphibian hosts, a situation likely to be occurring in the field where different types of selective pressures may act together to create a diverse panel of closely related evolving strains of fast replicating viruses. Such ranavirus diversity may be partly responsible for the variability in mortality events in natural populations of amphibians (Lesbarrères et al. 2012). One word of caution is however needed. The work above is based on water bath (immersion) infections using 10 000 PFU/mL. Plaque assays, used to determine infectious virus titer, show some level of inherent variability and thus differences (especially small ones), even if statistically significant, may reflect variation in actual multiplicities of infection. Thus, although variation in viral input may not impact assessment of host genotype, host species, and temperature, it may impact assessment of relative levels of virulence.

The diversity of ranavirus strains present in nature may constitute a framework for resistance to evolve in amphibian populations. Such a coevolutionary scenario is supported by reports of a wide range of susceptibility in amphibian hosts. Several North American studies have indicated that NLF and WF are particularly sensitive to ranavirus while bullfrogs are relatively resistant (Hoverman et al. 2011). Our results showed significant differences in ranavirus susceptibility between NLF and WF as well as among WF clutches. WF tadpoles presented higher mortality and infection probabilities than NLF tadpoles but the amplitude of this difference changed with the type of virus strain, suggesting the existence of species × strain interactions. For instance, although NLF tadpoles were generally more resistant and tolerant to infection than WF tadpoles, they were particularly sensitive to wt-FV3 while azacR was particularly efficient at infecting WF. We also observed host genotypes × strain interactions where WF1 and WF2 differed significantly in their susceptibility to infection and showed different probability of death. For instance, WF1 tended to have a higher probability of death when exposed to either azacR or SsMeV while WF2 highest mortality was associated with azacR and wt-FV3. Both species × strain and host genotype × strain interactions were significant drivers of infection in our experiment but they differed in the intensity of their effects. Generally, we observed a stronger and more consistent effect of “species” as compared to “genotypes” suggesting that a gradient of genetic distances among hosts, ranging from phylogenetic to genotypic, can result in a range of different infection outcomes. While we cannot exclude the possibility that some egg masses used in our experiment had different degrees of genetic variability due to multiple paternities, it seems unlikely that this has led to susceptibility differences because experimental evidence suggests that the life-history traits of offspring resulting from multiple paternities and single paternity clutches do not differ significantly in amphibians (Byrne and Roberts 2000).

### Reaction norms in response to temperature

While genotype × genotype interactions represent a functional explanation for variable mortality in the ranavirus-amphibian system, our results also suggest that temperature modulates such interactions and further determines outcomes. Temperature can modulate the host’s ability to mount an effective immune response (Lazzaro and Little 2009). For example, increasing temperature can induce a better antibody response in *Xenopus* (Wabl and Du Pasquier 1976) and promote a more efficient T cell response as evidenced by faster skin graft rejection (Robert et al. 1995). In contrast, decreasing temperature has been documented to induce host immunosuppression and enhance infection in several systems such as winter saprolegniosis in channel catfish (Bly et al. 1993). In the context of our experiment, both mortality and infection probabilities were significantly higher under colder conditions, suggesting that the metabolic activity of the host and particularly its immune defenses may have been depressed with a decrease in temperature. However, cold temperatures were more detrimental to NLF tadpoles than to WF tadpoles. The wood frog is the most northern frog species in North America. It experiences a wide array of environmental conditions (e.g., temperature fluctuations), exhibits extensive phenotypic plasticity (Herreid and Kinney 1967; Berven 1982), and presents specific biochemical adaptations that help it survive freezing temperatures (Storey and Storey 1984). These specific adaptations to cold temperatures make the wood frog particularly adapted to deal with a significant decrease of temperature and may be responsible for the species–temperature interactions we observed.

Similarly, virus strains associated mortality and infection probabilities differed depending on the temperature. The higher probability of death associated with wt-FV3 infection as compared to the other strains was striking under warm conditions but the differences disappeared in cold conditions. Likewise, azacR was associated with a higher infection probability in warm conditions only, illustrating strain-specific reaction norms. Most of the documentation available regarding reaction norms in pathogen infectivity come from *in vitro* studies. For instance, temperature can regulate the kinetics of virus replication by influencing the rate of viral protein and nucleic acid synthesis (Stairs 1978). In general, lower temperature inhibits virus replication and infection is limited, while virus reproduction within the host increases with temperature until a particular threshold (Ghosh and Bhattacharyya 2007). This pattern has been documented in several virus genera including baculoviruses, NPV (nuclear polyhedrosis virus; Ribeiro and Pavan 1994) and WSSV (white spot syndrome virus; Du et al. 2006, 2008). In the case of ranaviruses, FV3 isolated from frogs in the United Kingdom grows *in vitro* between 8°C and 30°C with slower replication below 15°C and the fastest replication observed at 30°C. In salamanders, the ranavirus ATV (*Ambystoma tigrinum* Virus) replicates very rapidly at 26°C, more gradually at 18°C, and very slowly at 10°C (Rojas et al. 2005). Preliminary data on *in vitro* replication rate of the three strains used in this experiment suggest that wt-FV3 reaches maximum replication rates at 30°C while azacR and SsMev replicate best at 22°C and 14°C respectively (P. Echaubard, A. Ferreira Lacerda, D. Lesbarrères and C. R. Brunetti, unpublished data), confirming strain-specific replication optimums and supporting some of the temperature-dependent patterns of infection outcomes observed in this experiment (e.g., wt-FV3 being particularly virulent in warm conditions). Together with host × strains, and host × temperature interactions, these strain × temperature interactions indicate strong synergies among ranavirus disease drivers and demonstrate that ranavirus-associated mortality and infection probability are not only contingent upon the specific infecting strains but also upon the host species/genotype and the temperature under which the interaction occurs.

### Applications in the context of emerging infectious diseases

Two practical aspects emerge from our work. First, the occurrence of species × strains and host genotype × interactions suggests that host phylogeny and genotype are key parameters influencing virus-associated mortality, highlighting the role of coevolutionary processes in defining amphibian–ranavirus interactions. In particular, genotype-specific interactions in this system indicate that a potentially large number of resistance and/or virulence alleles can accumulate in amphibian and ranavirus populations through balancing selection (Burdon and Thrall 2009). In natural populations, rapid evolution of resistance traits may allow hosts to respond to strong selective pressure from pathogens and to naturally buffer disease severity. However, changing habitats can alter barriers to transmission among species (Miller et al. 2014), reduce host population size and genetic diversity, and in turn lead to more common outbreaks of new or generalist strains of pathogens (Daszak et al. 2000). It is therefore critical that conservation programs acknowledge the role of coevolutionary processes between host and pathogens in determining infection outcomes and focus on maintaining gene flow among host populations in order to minimize genetic drift and the loss of resistance alleles (Altizer et al. 2003). From a community ecology perspective, if certain host species (e.g., reservoir species) responsible for amplifying ranaviruses persist or thrive, then disease transmission and risk may increase. On the other hand, if these species become rare, or disappear, disease risk may decrease. There is evidence that species-poor assemblages are usually dominated by susceptible host species (Johnson et al. 2013) and that maintaining high degree of species diversity is critical to dilute disease risk and minimize pathogen transmission because density of reservoir species are down-regulated (Johnson et al. 2012). Thus, maintaining high species diversity and limiting the proliferation of less susceptible (reservoir) species are key elements to consider when integrating disease risk in conservation practices. Future studies should thus better delineate host–pathogen specificities, document geographic strains, and characterize environmental, spatial, and temporal fluctuations associated with disease dynamics.

Second, our study documents the strong influence of temperature in increasing variability in mortality and infection through host and/or strain × temperature interactions. In the current context of global warming where temperature is expected to fluctuate widely (Altizer et al. 2013), our results suggest a strong potential for unexpected mortality and infection dynamics since not only host biology would be affected but pathogens replication and ultimately their virulence may be modulated. It appears therefore important to detect and isolate ranavirus strains from the field and to investigate their thermal optimums in both laboratory and natural settings in order to determine windows of disease risk associated with changing temperature patterns.

## Conclusion

Ranaviruses have been involved in epizootics and mass die-offs of amphibian populations identifying them as a potentially strong source of selection in this system (Collins and Storfer 2003; Teacher et al. 2009; Miller et al. 2011). Our study suggests that the variability of ranavirus-associated mortalities in amphibians may be due to specific genetic interactions where a given host responds differently to different ranavirus strains and vice versa, in turn suggesting the potential for coevolution in this system. These specific interactions are further influenced by the temperature at which amphibian–ranavirus interactions occur, resulting in mortality patterns that are difficult to predict. Moreover, this variability is likely to be larger under natural conditions where other environmental variables such as resource availability (Bedhomme et al. 2004) and developmental stage (Johnson et al. 2011) are important. Environmental heterogeneity significantly increases the difficulty of predicting host and pathogen fitness-related trait variation as the environment may alter both selection specificity and strength (Lazzaro and Little 2009; Wolinska and King 2009), leading to variation in specific gene frequencies in nature. We therefore suggest that using an eco-evolutionary framework will be more successful in explaining changes in gene frequencies as a response to selective forces in amphibian-ranavirus systems (Cousyn et al. 2001; Mitchell et al. 2005), will lead to a better understanding of the epidemiology and evolution of wildlife diseases and will guide innovative and efficient conservation strategies, especially in the current context of climate change (Lesbarrères et al. 2012; Daskin and Alford 2012).

## Data archiving

Data for this study are available from the Dryad Digital Repository: http://dx.doi.org/10.5061/dryad.qp544.
